# Comprehensive performance and optimization of micro textured slipper pair of axial piston pumps

**DOI:** 10.1038/s41598-025-97669-x

**Published:** 2025-04-11

**Authors:** Ping Xu, Jing Luo, Yinghua Yu, Jiaxing Shen, Wenli Li

**Affiliations:** College of Mechanical Engineering, Liaoning Technical University, Fuxin, 123000 China

**Keywords:** Axial piston pump, Slippers, Micro-texture, Friction and wear, Temperature, Multi-objective optimization, Mechanical engineering, Aerospace engineering

## Abstract

The present study aims to fully exploit the potential of micro-texturing to increase the operational efficiency of piston pump slipper pairs and expand the repertoire of discretely distributed pit micro-textures. Four micro-textures, each featuring unique opening and pit configurations, have been proposed. These micro-textures are applied to the working surface of the axial piston pump slipper to optimize friction and wear characteristics, mitigate heat generation during operation, and decrease leakage within the slipper pair. The influence of micro-texturing on the lubricant oil film of the slipper surface was evaluated using computational fluid dynamics (CFD) and experimental methodologies. The research employed response surface methodology to examine the impact of the position distribution and shape size parameters of the micro-textures on the bearing pressure, friction coefficient, temperature, and oil film leakage between slipper pairs, followed by multi-objective and multi-parameter optimization. The results indicate that the implementation of micro-textures substantially enhances the operational performance of the slipper surface. A texture design that integrates an elliptical opening with an elliptical offset parabolic pit body situated at the innermost two-ring support band of the slipper is recommended. Compared with the original prototype, this optimized design yields a 19.69% increase in the bearing pressure and a 21.08% reduction in the friction coefficient, coupled with a 14.20% decrease in the average temperature and a 14.03% reduction in oil film leakage. This research provides a valuable reference for the design of a wider array of pit micro-texture geometries and offers theoretical support for the performance enhancement of axial piston pumps.

## Introduction

Micro-texture technology employs a diverse array of technical methods to manipulate microstructures on the surfaces of objects. These microstructures typically adhere to specific arrangement patterns, and their presence enhances the surface properties. In 1966, Hamilton DB et al.^1^ pioneered the use of surface texturing to enhance the surface friction characteristics of mechanical components. Subsequently, numerous researchers have delved into the enhancement of working surface performance through surface micro-texturing. Piston pumps, with a history of more than 110 years, have been widely adopted in sectors such as petrochemicals, aerospace, rail transportation, military, agriculture, and shipbuilding, owing to their consistent flow, high efficiency, robust self-priming capabilities, favorable power density ratio, and extensive application spectrum. Throughout this period, the slipper pair has remained one of the pivotal friction pairs that dictates the operational performance of the piston pump. Currently, the piston pump is evolving toward higher speeds, increased pressures, larger flow rates, and extended lifespans^2^. To accommodate and fulfill these developmental requirements, investigating the enhancement of piston pump performance by incorporating micro-textures into the slipper pair is of significant theoretical and practical importance. This has emerged as a focal point of research for scholars in the field.

Kumar S et al.^3^ studied the effects of size and position parameters of groove micro-textures on the load pressure and leakage of the slipper pair oil film. The results demonstrated that the presence of micro-textures significantly affects the load-bearing and stress of the slipper pair oil film. Zhao JA et al.^4^ reported that the surface micro-texturing of piston pump friction pairs enhances the bearing capacity of the oil film. Furthermore, this study corroborated the perspective that micro-textures can lower the surface friction coefficient by trapping lubricating oil and wear debris^5,6^. Ye SG et al.^7^ examined the effects of micro-texture on the shear force and bearing capacity of oil films on slipper surfaces. Their research was conducted across various rotational speeds and load pressures. They reported that when the area density of the micro-texture is 24% and the ratio of pit depth to diameter is 0.3, the oil film on the slipper surface achieves a high bearing capacity under elastohydrodynamic lubrication conditions. Ramesh A et al.^8^ has machined micro textures on stainless steel surfaces, with the dimensions of these micro textures ranging from 28 to 257 μm. Through simulation and experimental studies, it has been found that under fully lubricated conditions, the friction coefficient of surfaces with micro textures is reduced by 80% compared to those without textures. This demonstrates that micro textures can effectively improve the wear resistance of friction pairs. Jiang J et al.^9^ designed three types of microstructures for the slipper pair to improve lubrication performance: micro chamfer, micro fillet, and micro step. Through simulation and experimental research methods, it was found that all three types of microstructured slipper significantly improved lubrication performance. Subsequently, in order to more accurately predict and improve the lubrication characteristics of the slipper/pad interface, a lubrication numerical model and algorithm^10^ were proposed, with the optimal micro-chamfer width and depth being 1.2 mm and 3.5 μm, respectively. The experimental values were basically consistent with the simulation values, verifying the correctness and effectiveness of the lubrication numerical model, and providing direction for further design of axial piston pumps.

Generally, pertinent research has demonstrated the feasibility of applying surface texture technology to the surfaces of slipper pairs to increase their performance. However, prior studies have focused predominantly on one or two aspects, such as the oil film bearing pressure, friction coefficient, temperature, and other properties of the slipper pairs. The shapes of micro-textures are typically circular, triangular, and square openings, whereas the cutoff shapes are semicircular, rectangular, and triangular, which to some extent limits the potential of micro-textures.

In previous research experience, an Elliptic Opening Offset Parabola Micro Texture (abbreviated as EOOPT)^11^ has been proposed. In this study, additional micro textures composed of different opening and pit shapes are proposed. They are respectively: the Elliptical Open Triangle Pit Micro Texture (abbreviated as EOTT), Circular Open Spherical Pit Micro Texture (abbreviated as COST), and Circular Open Triangle Pit Micro Texture (abbreviated as COTT). Through the application of theoretical analysis, simulation analysis, and experimental research, the optimal texture type and distribution scheme are identified. Furthermore, the effects of texture characteristic parameters on the bearing capacity, friction resistance, wear resistance, temperature increase, and sealing performance of the oil film in the slipper pair of an axial piston pump are examined. These parameters are optimized via multi-objective and multi-parameter optimization design theory, providing a valuable reference for enhancing the operational performance of axial piston pumps.

## Prototype selection and comprehensive performance analysis of slipper pairs

### Prototype selection

**Fig. 1:**
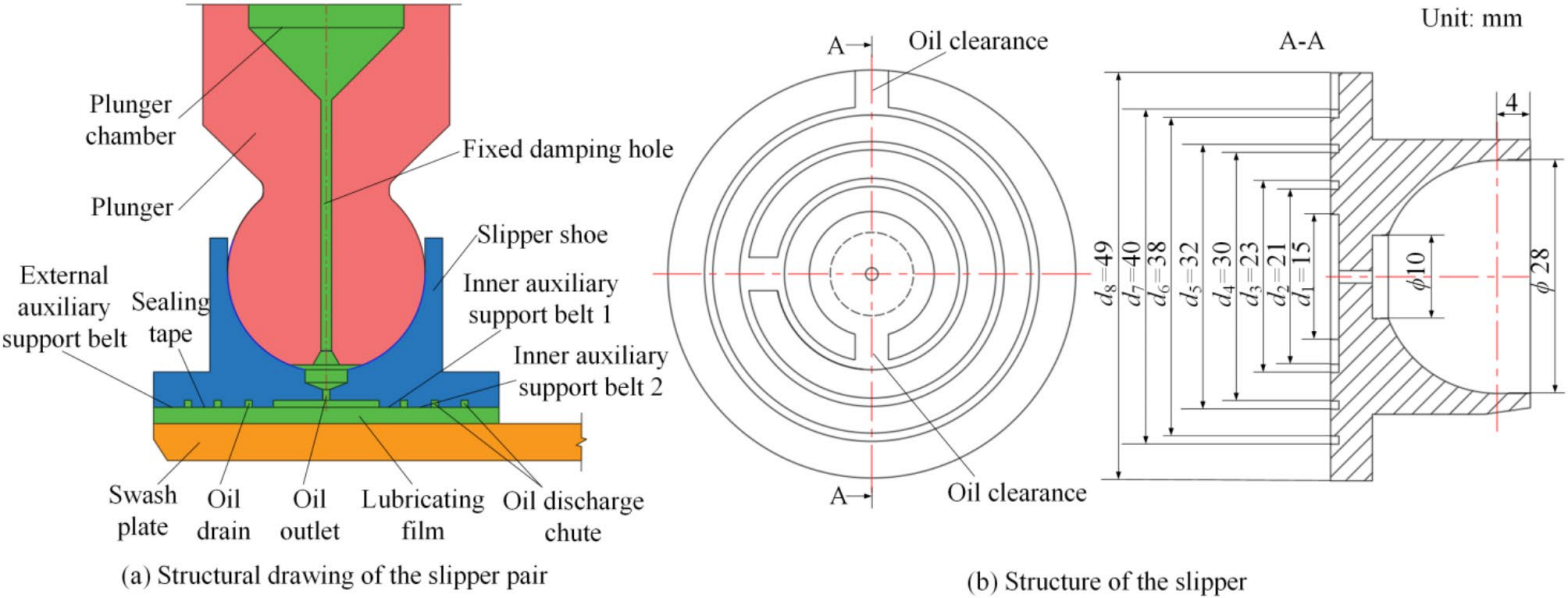
Prototype slipper pair and slipper structure diagram.

A swash plate axial piston pump was chosen as the prototype for this study. The configuration of the slipper pair is depicted in Fig. 1(a), whereas the detailed structure of the slipper itself is illustrated in Fig. 1(b). The bottom surface of the axial piston pump slipper features four distinct ring zones. Proceeding from the interior outward, these belts include internal auxiliary support belt 1, internal auxiliary support belt 2, a sealing belt, and an outer auxiliary support belt. Annular oil grooves are present between the support bands and the sealing band. Each support belt includes an oil gap, ensuring that the oil pressure within the inner oil chamber matches that of the inner oil tank, similarly, the pressure in the outer oil tank is equal to the ambient pressure.

### Motion analysis

**Fig. 2:**
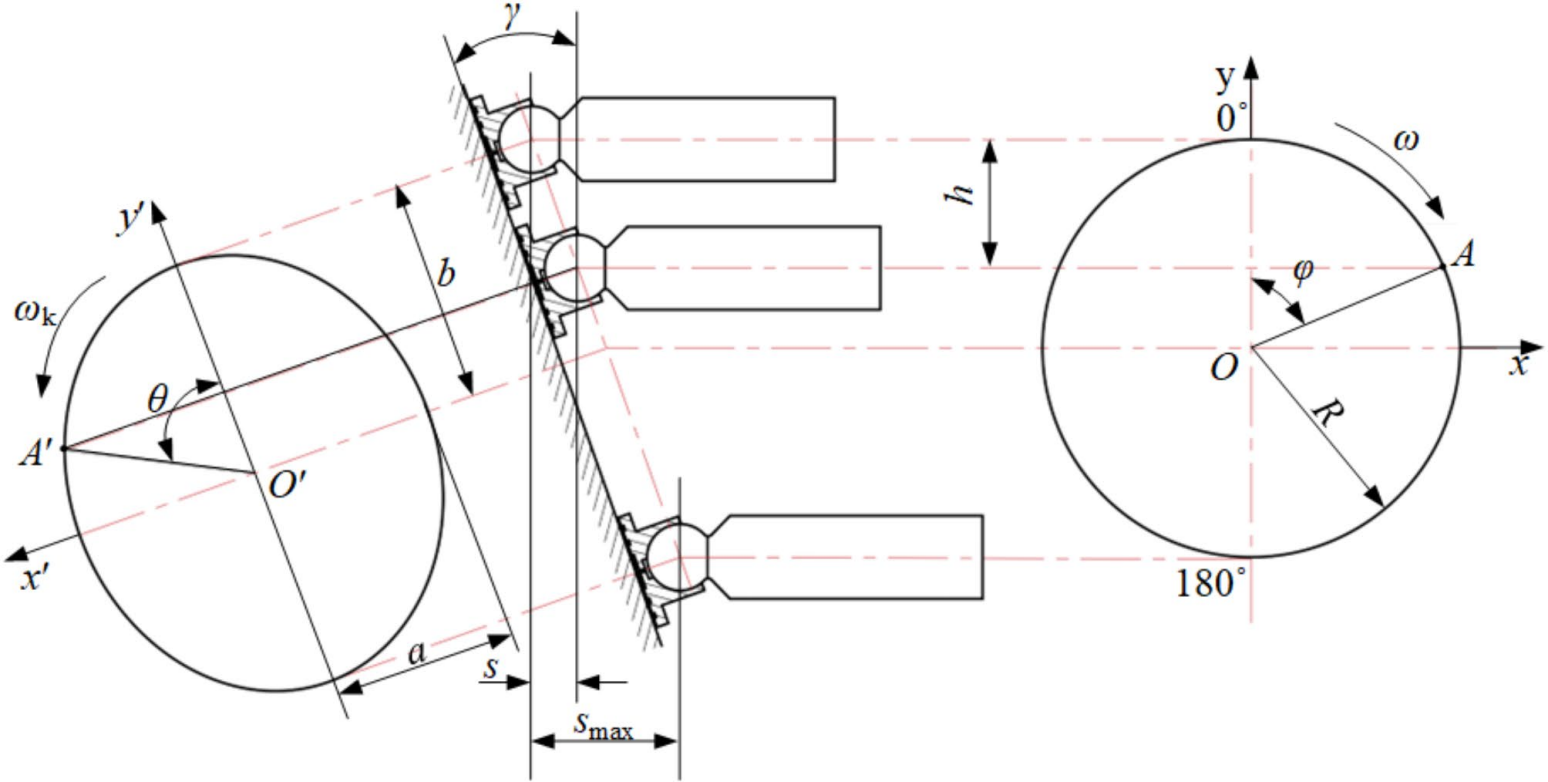
Motion analysis diagram of swash plate axial piston pump.

According to the operational principle of the piston pump, the axial piston motion analysis diagram, which includes the depiction of slippers, is presented in Fig. 2.

Upon establishing the positive orientation of the y-axis as the initial point of motion and with the swash plate’s clockwise rotation through an angle of *φ*, the axial displacement of the slipper from the initial point to point *A* is specified as follows:

1 $$s=R(1 - \cos \varphi )\tan \gamma$$

where *R* is the distribution circle radius of the plunger cavity on the cylinder block, mm, and *γ* is the inclined angle of the swash plate, °.

For Eq. (1), find the first-order guide, and obtain the movement speed of the plunger in the axial direction *v* :

2 $$v=\frac{{ds}}{{dt}}=\omega R\sin \varphi \tan \gamma$$

where *v* is the plunger axial movement speed, m/s, and *ω* is the swashplate rotation angular velocity, rad/s.

The coordinates of the central point of the slipper ball socket *A*´ on the swash plate *O*´*x*´*y*´plane are as follows^12^:

3 $$\left\{ {\begin{array}{*{20}{c}} {x=R\sin \varphi } \\ {y=\frac{R}{{\cos \gamma }}\cos \varphi } \end{array}} \right.$$

At this point, the tangential velocity *v*_1_ of point *A*’ relative to the rotation of the swash plate axis is^12^:

4 $${v_1}=\sqrt {{{\left( {\frac{{dx}}{{dt}}} \right)}^2}+{{\left( {\frac{{dy}}{{dt}}} \right)}^2}} =R\omega \sqrt {1+{{\sin }^2}\varphi \left( {\frac{{1 - {{\cos }^2}\gamma }}{{{{\cos }^2}\gamma }}} \right)}$$

### Simulation analysis of performance of prototype slipper pair

Suppose that the lubricating oil is an incompressible fluid, satisfying the following fluid motion N–S equation^13^:

5 $$\nabla \cdot \left( {\rho u\overrightarrow V } \right)= - \frac{{\partial p}}{{\partial x}}+\eta [(\frac{4}{3}\frac{{{\partial ^2}u}}{{\partial {x^2}}}+\frac{{{\partial ^2}u}}{{\partial {y^2}}}+\frac{{{\partial ^2}u}}{{\partial {z^2}}})+\frac{1}{3}(\frac{{{\partial ^2}v}}{{\partial x\partial y}}+\frac{{{\partial ^2}w}}{{\partial x\partial z}})]$$

6 $$\nabla \cdot \left( {\rho v\overrightarrow V } \right)= - \frac{{\partial p}}{{\partial y}}+\eta [(\frac{{{\partial ^2}v}}{{\partial {x^2}}}+\frac{4}{3}\frac{{{\partial ^2}v}}{{\partial {y^2}}}+\frac{{{\partial ^2}v}}{{\partial {z^2}}})+\frac{1}{3}(\frac{{{\partial ^2}u}}{{\partial y\partial x}}+\frac{{{\partial ^2}w}}{{\partial y\partial z}})]$$

7 $$\nabla \cdot \left( {\rho w\overrightarrow V } \right)= - \frac{{\partial p}}{{\partial z}}+\eta [(\frac{{{\partial ^2}w}}{{\partial {x^2}}}+\frac{{{\partial ^2}w}}{{\partial {y^2}}}+\frac{4}{3}\frac{{{\partial ^2}w}}{{\partial {z^2}}})+\frac{1}{3}(\frac{{{\partial ^2}u}}{{\partial z\partial x}}+\frac{{{\partial ^2}v}}{{\partial z\partial y}})]$$

where $$\overset{\lower0.5em\hbox{$\smash{\scriptscriptstyle\rightharpoonup}$}}{V}$$ is the lubricating oil velocity vector, $$\overset{\lower0.5em\hbox{$\smash{\scriptscriptstyle\rightharpoonup}$}}{V} = u\hat{i} + v\hat{j} + w\hat{k}$$; *u*,* v*,* w* are the components of the lubricating oil velocity in the *x*, *y*, and *z* directions, respectively, m/s; $$\hat {i},\hat {j},\hat {k}$$ are the unit vectors in the *x*, *y*, and *z* directions, respectively; *p* is the lubricating oil pressure, Pa; *η* is the dynamic viscosity of the lubricating oil, Pa s; and *ρ* is the lubricating oil density, kg/m^3^.

When thermal radiation is not considered, the viscous fluid satisfies the energy equation as follows^13^:

8 $$\rho \frac{{d\left( {{c_p}T} \right)}}{{dt}}=\nabla \cdot \left( {{\lambda _0}\nabla T} \right) - \frac{T}{\rho }\frac{{\partial \rho }}{{\partial T}} \cdot \frac{{dp}}{{dt}}+\Phi$$

where *c*_p_ is the specific heat at constant pressure, J/(kg °C); *T* is the fluid temperature, K; *λ*_0_ is the thermal conductivity of the lubricating oil, W/(m °C); and Φ is the dissipation work, W.

The pressure at each point on the lubricating oil film is integrated to obtain the bearing capacity of the lubricating oil film *N* as^11^:

9 $$N=\iint\limits_{\Omega } {p\left( {x,y} \right)dxdy}$$

where Ω is the contact area of the whole distributed pressure area, mm^2^.

The shear stress at each point on the lubricating oil film is integrated to obtain the friction *F*_f_ produced by the lubricating oil film on the surface of the slipper^11^:

10 $${F_f}=\iint\limits_{\Omega } {\eta \frac{{\partial u}}{{\partial z}}}dxdy$$

The friction coefficient *µ* is as follows^11^:

11 $$\mu =\frac{{{F_f}}}{N}=\frac{{\iint\limits_{\Omega } {\eta \frac{{\partial u}}{{\partial z}}dxdy}}}{{\iint\limits_{\Omega } {p\left( {x,y} \right)dxdy}}}$$

The average temperature of the oil film between the slipper pairs *T*_TB_ is as follows^15^:

12 $${T_{TB}}=\frac{{\iint\limits_{\Omega } {T(x,y)dxdy}}}{S}$$

where *T*(*x*, *y*) is the temperature of each point of the lubricant oil film, K, and *S* is the area of the oil film, mm^2^.

The average flow velocity of the oil film is integrated into the area to obtain the leakage amount *Q*_total_ as^16^:

13 $${Q_{total}}=\iint\limits_{s} {\mathop V\limits^{ \to } dxdy}$$

**Fig. 3:**
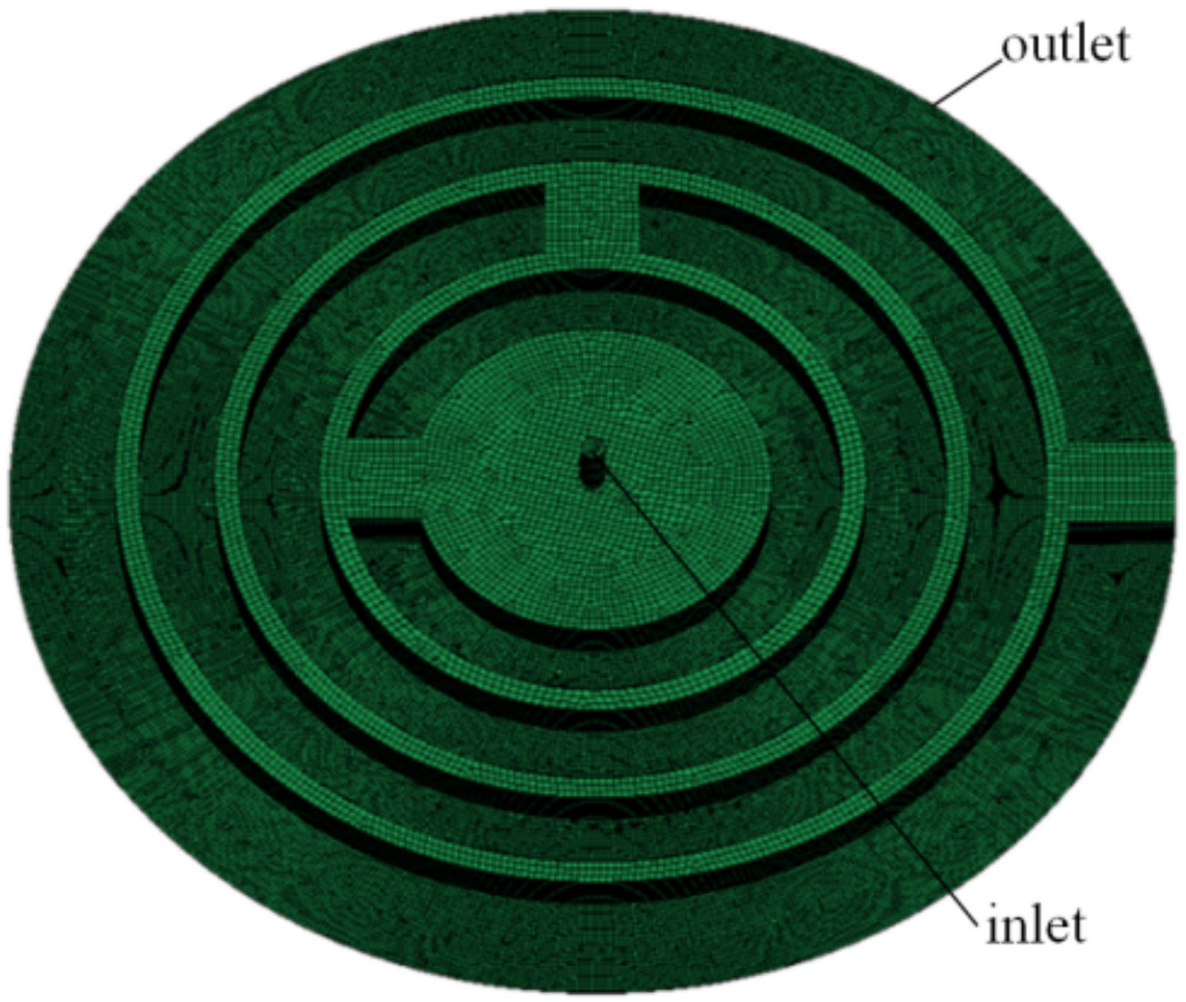
Lubricating oil film grid diagram.

For the oil film simulation of the whole slipper pair, a three-dimensional model of the oil film between the slipper pairs is established on the basis of its structural characteristics. The oil film thickness was set to 30 μm^14^. Owing to the small thickness of the oil film, a hexahedral mesh that can provide high spatial resolution was selected to mesh the model. The results are shown in Fig. 3. The simulation conditions are set in Fluent as follows: the liquid flow state is set to laminar flow, the separate solver for the solution is selected, the inlet pressure is 31.5 MPa, the outlet pressure is 0.1 MPa, the initial temperature is 50 °C, the contact surface of the oil film and the slipper is set to the stationary surface, the surface in contact with the swash plate is set to the rotating wall with a speed of 1500 r/min, the lubricating oil density *ρ* is 872.5 kg/m^3^, and the lubricating oil dynamic viscosity *η* is 1.553 Pa s.

**Fig. 4:**
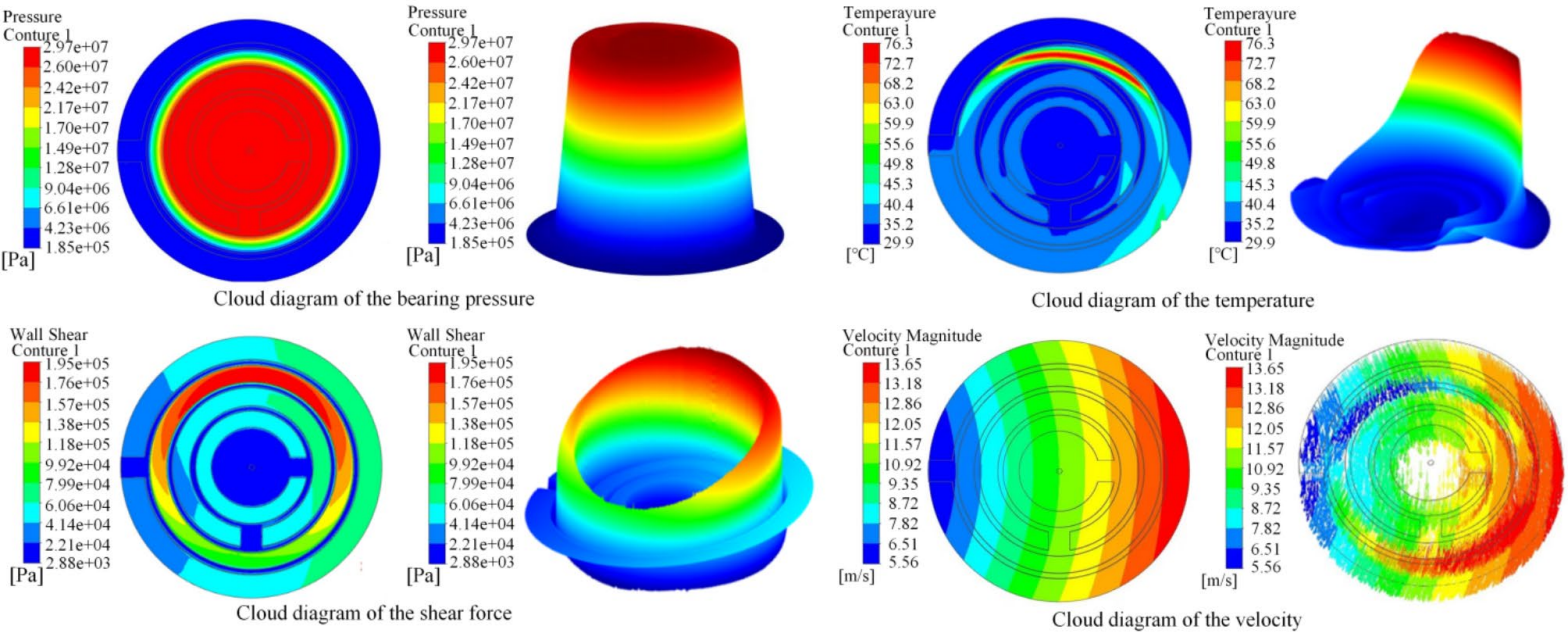
Oil film cloud diagram of the prototype slipper pair.

Through the above simulation analysis, the bearing pressure, shear force, temperature, and velocity nephograms of the oil film for the prototype slipper pair are obtained, as depicted in Fig. 4.

The maximum pressure of the oil film is concentrated in the inlet area of the oil chamber and decreases successively along the outlet direction. The oil-bearing pressure at the sealing belt decreases significantly, and the shear force distribution is concentrated, forming a local high-temperature area on the surface. The leakage velocity is related to the rotational motion of the oil film. During the process of liquid motion, the high-speed area and the low-speed area of leakage are approximately circumferentially symmetrical due to inertia. These results are consistent with the working characteristics of the oil film slipper pair. Through Eq. (9) to (13), the bearing pressure, friction coefficient, average temperature, and leakage amount of the oil film of the prototype axial piston pump are 13,710,337 Pa, 0.00446, 52.8 °C, and 0.0173 L.s^-1^, respectively.

### Determination of micro-texture unit pits

**Fig. 5:**
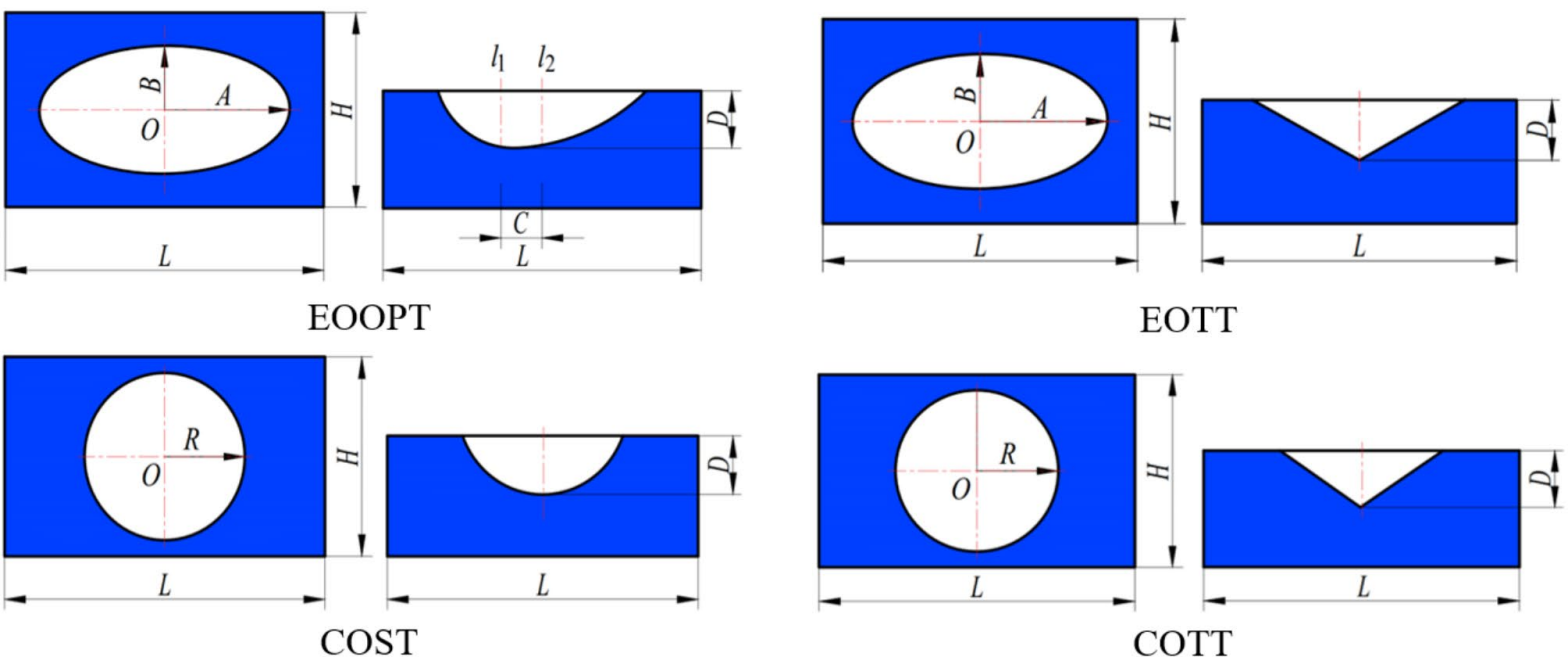
Shapes of four candidate micro-units.

At present, the hole shapes of discrete pit units are predominantly spherical pits^15^. To enhance the study of micro-texture cells, as previously mentioned, this paper proposes four types of micro-textures. Their opening shapes encompass ellipses and circles, whereas the pit body shapes include ellipsoids, triangles, and ellipsoidal shapes with central offsets. To ensure comparability, the area texture rate was kept close to 15% and the maximum depth D was set at 55 μm^16^ when the micro-texture properties of different shapes were analyzed. The specific shape and size parameters of the micro-texture units are detailed in Table 1 and Fig. 5.

**Table 1:** Four kinds of micro-texture cell parameters.

Texture type	Size/µm	Texturing ratio/%	Texture type	Size/µm	Texturing ratio/%
EOOPT	*L* = 1200 μm; *H* = 800 μm *A* = 300 μm; *B* = 150 μm *C* = 40 μm; *D* = 55 μm	14.7	EOTT	*L* = 1200 μm; *H* = 800 μm *A* = 300 μm; *B* = 150 μm *D* = 55 μm	14.7
COST	*L* = 1200 μm; *H* = 800 μm *R* = 212 μm; *D* = 55 μm	14.7	COTT	*L* = 1200 μm; *H* = 800 μm *R* = 212 μm; *D* = 55 μm	14.7

**Fig. 6:**
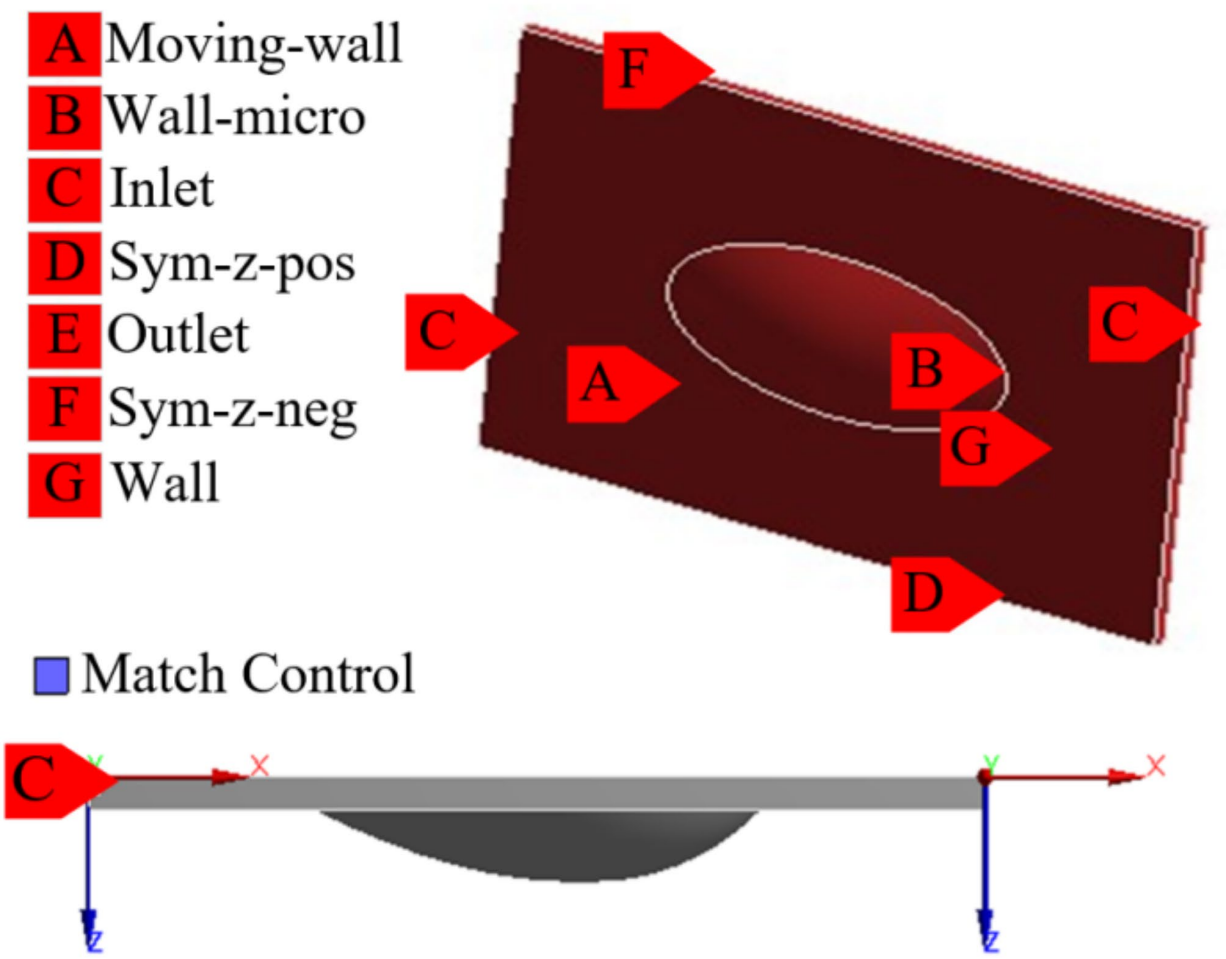
Micro-texture unit simulation setup diagram.

For the texture unit oil film simulation, the setup is depicted in Fig. 6. Given the micro-texture’s small overall dimensions, its rotational motion is approximated as linear. The diminutive size of the micro-texture results in a minor pressure differential between the inlet and outlet. Consequently, the inlet and outlet pressures are established at 0.2 MPa. Other simulation conditions, such as the choice of solver, the magnitude of the rotational speed, and the setting of lubricant parameters, are the same as in “Simulation analysis of performance of prototype slipper pair”.

**Fig. 7:**
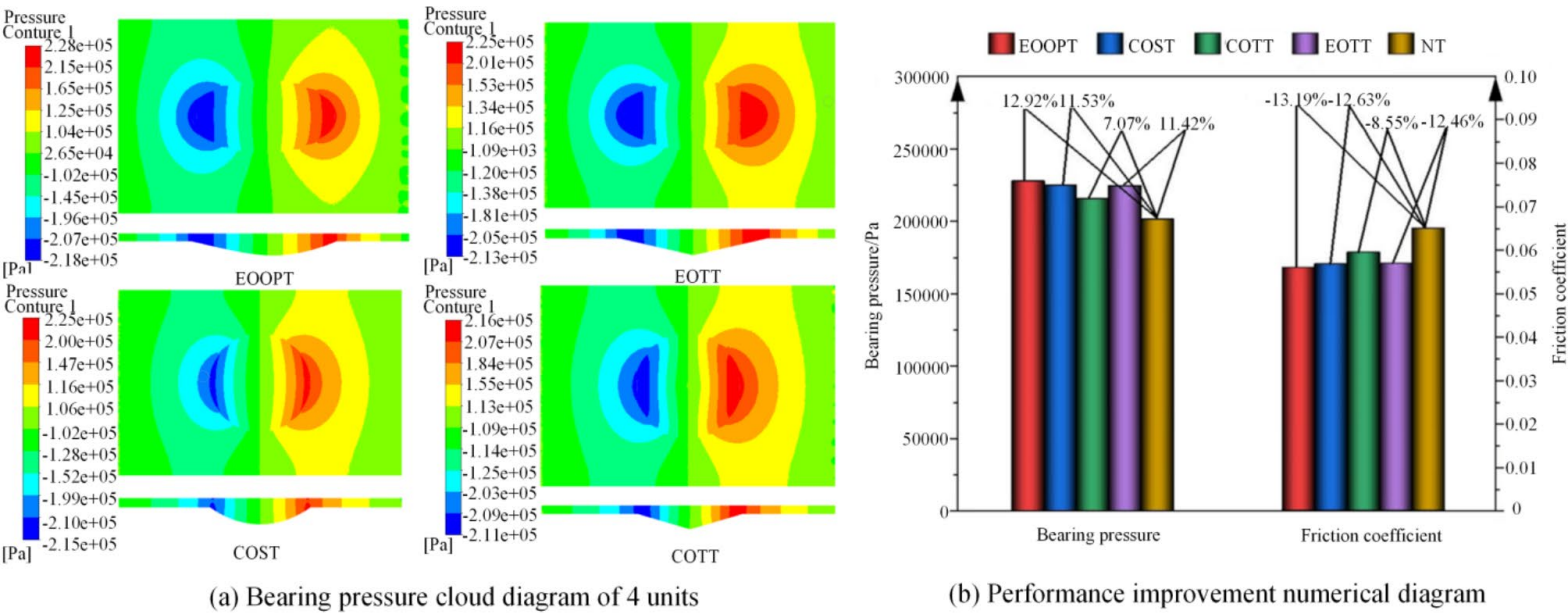
Comparison of the performance of the five kinds of micro-units.

The cloud maps of bearing pressure for four types of micro-textured units and the numerical comparison of bearing pressure and friction coefficient between the four types of micro-textured units and the prototype (No micro-texture, abbreviated as NT) are shown in Fig. 7. The oil film on the textured surface demonstrated a degree of improvement in bearing pressure relative to the untextured oil film. The sequence of effectiveness in enhancing the bearing pressure and reducing friction is as follows: This is attributed to the fact that the elliptical opening is more advantageous for liquid inflow than the circular opening is, and the ellipsoidal pit body is more conducive to the generation of fluid dynamic pressure than the spherical and triangular pit bodies are while also alleviating the stress concentration within the pit body. Therefore, the combination of the elliptical opening with the ellipsoidal pit body yields a superior bearing effect. Consequently, the texture shape selected for application on the slipper surface is EOOPT.

## Microtextured slipper scheme

**Fig. 8:**
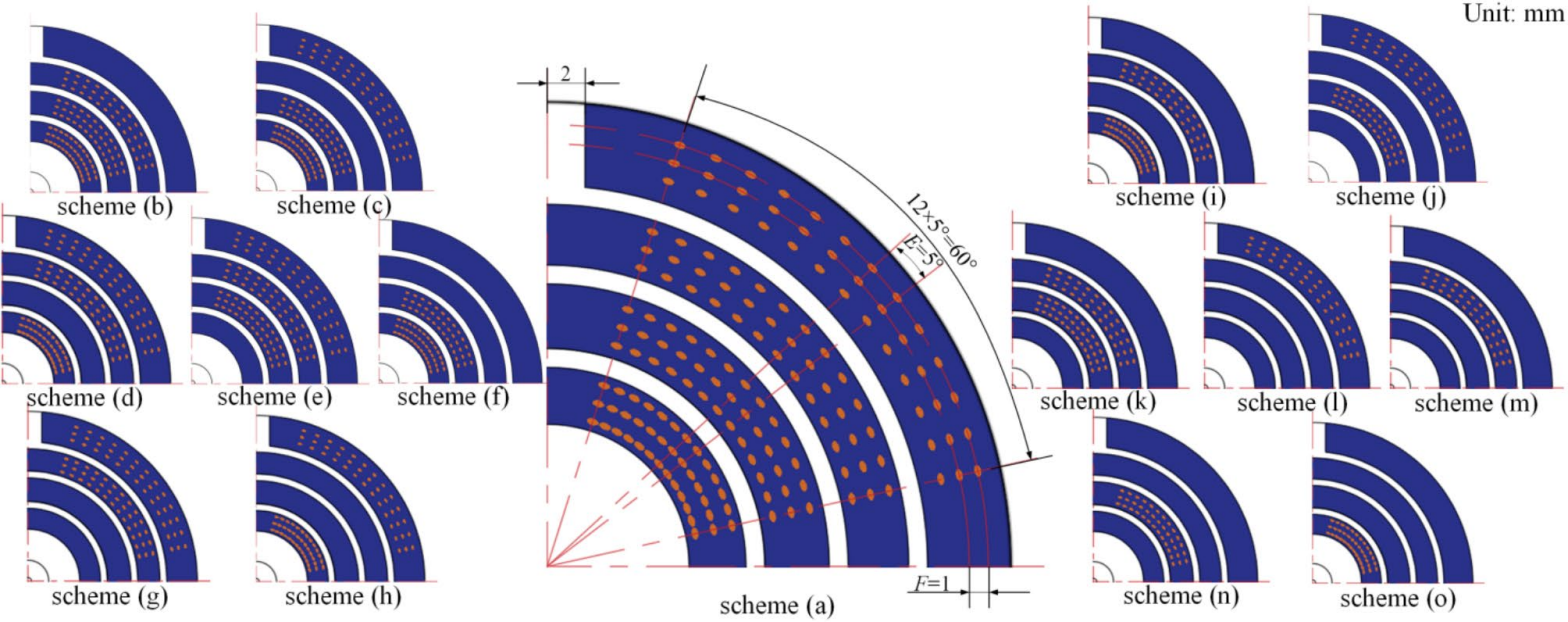
Texture distribution map.

As previously mentioned, there are four slippers and swash plate contact ring banks: inner auxiliary support belt 1, inner auxiliary support belt 2, the sealing belt, and the outer auxiliary support belt. There should be a total of 15 texture schemes for this purpose, which are named as follows: the four ring banks are textured as scheme (a), whereas only the outer auxiliary support belt, the sealing belt, the inner auxiliary support belt 2, and the inner auxiliary support belt 1 are textured as schemes (b), (c), (d), and (e), respectively. The textures of the inner auxiliary support belts 1 and 2 are shown in scheme (f), the textures of the outer auxiliary support belt and the sealing belt are shown in scheme (g), the textures of the outer auxiliary support belt and the inner auxiliary support belt 1 are shown in scheme (h), the textures of the inner auxiliary support belt 1 and the sealing belt are shown in scheme (i), the textures of the outer auxiliary support belt and the inner auxiliary support belt 2 are shown in scheme (j), and the textures of the inner auxiliary support 2 and the sealing belt are shown in scheme (k). Schemes (l), (m), (n), and (o) are textured separately for the outer auxiliary support belt, the sealing belt, the internal auxiliary support belt 2, and the internal auxiliary support belt 1. The 1/4 structure of the 15 texture schemes on the surface of the slipper is shown in Fig. 8. The size parameters of EOOPT are initially set to be the same as the size parameters of “Simulation analysis of performance of prototype slipper pair”. Taking the 1/4 ring bank as an example, a 60° fan-shaped texture array distributed along the circumferential direction is constructed. The central angle *E* of the two adjacent micro-texture centers in the radial direction is set to 5.0°, and the radial distance *F* between the adjacent two textures is set to 1 mm.

The mesh size for all micro-texture areas is set to 20 μm, and the remaining simulation conditions are consistent with those in “Simulation analysis of performance of prototype slipper pair”. The calculation results are presented in Table 2. Of the 15 schemes, scheme a has the best effect on improving the oil film temperature, and scheme f has the best effect on improving the oil film’s load-bearing pressure, friction coefficient, and leakage amount. In practical applications, the inner auxiliary support belts 1 and 2 are connected to the oil chamber, forming a high-pressure area due to the oil gap. Excessive micro-structure processing may lead to a reduction in the local strength of the material, thereby diminishing its rigidity and mechanical strength. Considering the comprehensive performance requirements and economic principles, the texture position is ultimately selected as scheme (f).

**Table 2:** Performance of slipper pairs in different micro-texture schemes.

Scheme	*p×*10^6^/Pa	µ × 10^− 3^	T_TB_/°C	Q_total_×10^− 3^/L s^− 1^	scheme	*p×*10^6^/Pa	µ × 10^− 3^	T_TB_/°C	Q_total_×10^− 3^/L.s^− 1^
NT	13.71	4.46	52.80	17.30	(h)	15.81	3.62	46.79	15.45
(a)	15.70	3.66	45.68	15.85	(i)	15.77	3.68	46.51	15.85
(b)	15.68	3.67	46.43	15.84	(j)	15.80	3.65	46.82	15.76
(c)	15.71	3.64	46.67	15.25	(k)	15.66	3.66	46.52	15.84
(d)	15.65	3.62	46.45	15.81	(l)	14.93	3.79	47.86	15.99
(e)	15.88	3.68	46.46	15.83	(m)	14.91	3.78	47.81	15.98
(f)	15.91	3.60	46.31	15.24	(n)	14.95	3.71	47.86	15.94
(g)	15.69	3.63	46.55	15.89	(o)	14.98	3.73	47.82	15.90

## Optimization design of micro-texture feature parameters

Both the distribution parameters and size parameters of micro-texture significantly impact the tribological properties of textured friction pairs^17–25^. Consequently, the central composite design (CCD) feature of Design-Expert was employed to devise a six-factor, five-level response surface test scheme^26^to assess the effects of micro-texture parameters on the performance of slipper pairs. The test scheme and results are presented in Table 3.

**Table 3:** Performance of slipper pairs with different micro-texture schemes.

Order number	A/µm	B/µm	C/µm	D/µm	E/°	F/µm	*p×*10^6^/Pa	µ×10^− 3^	T_TB_/°C	Q_total_×10^− 3^/L s^− 1^	Order number	A/µm	B/µm	C/µm	D/µm	E/°	F/µm	*p×*10^6^/Pa	µ×10^− 3^	T_TB_/°C	Q_total_×10^− 3^/L s^− 1^
1	400	90	30	75	4	1100	15.81	3.75	47.62	15.23	21	400	210	50	35	6	900	16.12	3.63	45.76	14.85
2	200	90	50	35	6	1100	15.67	3.86	48.87	15.36	22	350	150	40	55	5	1000	16.06	3.57	45.55	15.00
3	200	90	30	35	6	900	15.63	3.79	47.78	15.27	23	400	210	50	35	4	1100	16.42	3.53	45.92	14.84
4	200	210	30	35	6	1100	15.81	3.73	46.61	15.05	24	300	120	40	55	5	1000	15.82	3.74	46.99	15.19
5	200	90	50	75	6	900	15.62	3.82	48.50	15.34	25	300	150	40	55	5	950	15.96	3.59	46.07	15.13
6	200	90	30	35	4	1100	15.61	3.83	48.59	15.35	26	300	150	40	55	5	1000	15.97	3.60	45.52	15.04
7	300	150	40	45	5	1000	15.87	3.66	46.56	15.06	27	400	90	50	35	4	900	15.81	3.76	47.61	15.21
8	200	210	50	75	6	1100	16.06	3.65	46.62	15.09	28	200	90	50	75	4	1100	15.60	3.79	47.74	15.28
9	200	210	30	75	4	1100	16.23	3.62	45.45	15.05	29	400	210	30	35	4	900	16.49	3.55	46.06	14.84
10	200	210	50	35	4	900	16.01	3.65	46.53	15.04	30	300	150	40	55	5.5	1000	15.97	3.64	45.80	15.01
11	400	90	30	75	6	900	15.71	3.79	47.69	15.25	31	300	150	40	55	5	1000	15.91	3.60	45.52	15.04
12	300	180	40	55	5	1000	16.08	3.64	45.67	15.01	32	300	150	40	55	5	1000	15.93	3.60	45.56	15.04
13	300	150	40	55	5	1000	15.91	3.60	48.63	15.04	33	200	90	30	75	4	900	15.77	3.82	47.98	15.31
14	200	210	30	75	6	900	16.07	3.65	46.98	15.08	34	300	150	40	65	5	1000	15.92	3.64	45.47	15.05
15	300	150	40	55	4.5	1000	15.99	3.62	45.57	15.02	35	300	150	45	55	5	1000	15.90	3.56	45.71	15.06
16	400	210	50	75	6	1100	16.45	3.53	47.96	14.75	36	400	210	50	75	4	900	16.61	3.50	46.67	14.83
17	250	150	40	55	5	1000	15.94	3.61	46.20	15.09	37	400	90	30	35	6	1100	15.69	3.85	48.35	15.28
18	300	150	40	55	5	1000	15.94	3.60	45.54	15.04	38	300	150	40	55	5	1000	15.95	3.60	46.83	15.04
19	400	90	50	75	6	1100	15.78	3.78	47.59	15.29	39	300	150	40	55	5	1050	15.94	3.58	46.00	15.14
20	300	150	35	55	5	1000	15.91	3.56	45.51	15.05	40	400	210	50	35	4	900	16.41	3.51	45.88	14.82

**Fig. 9:**
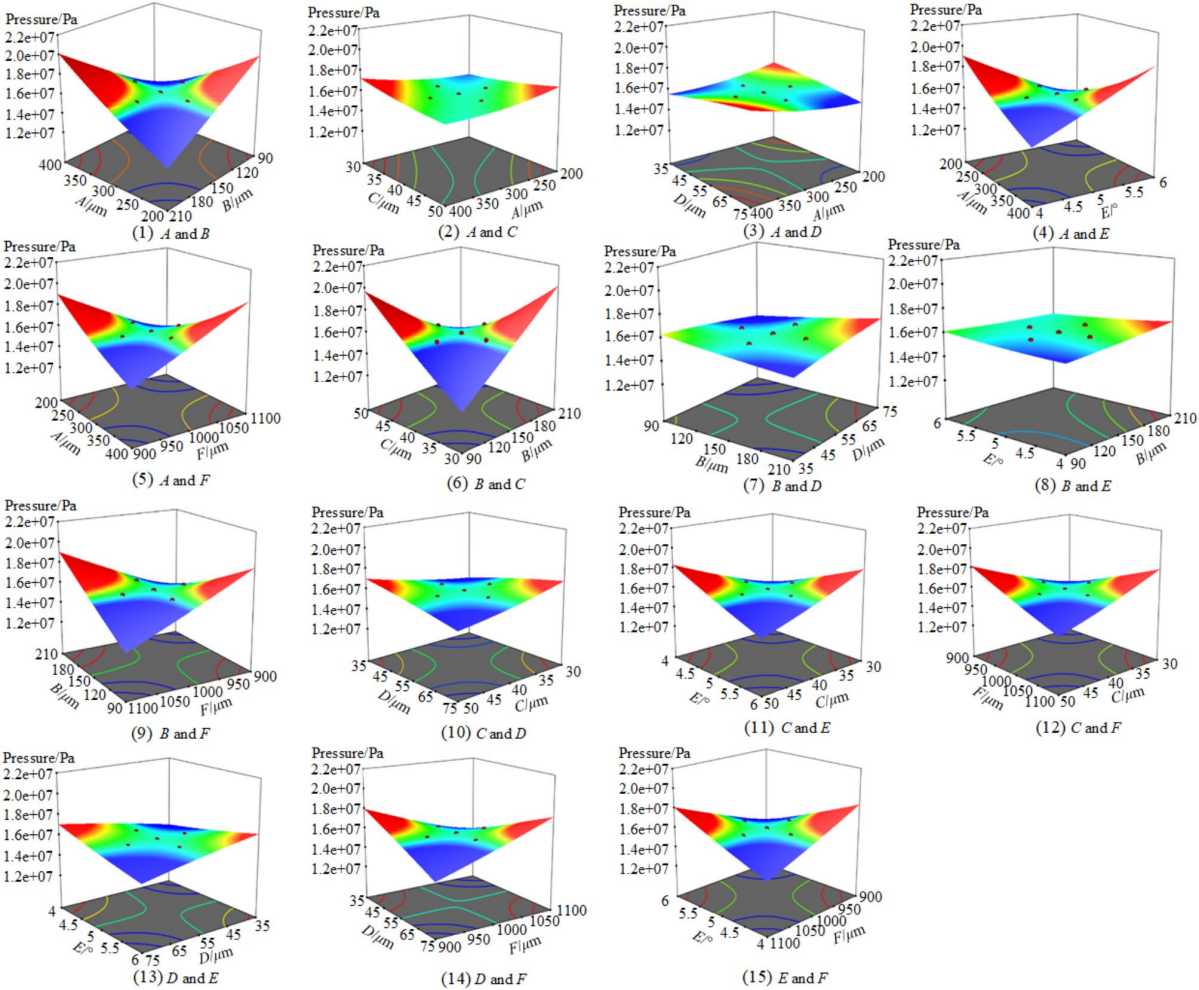
Response surface of the interaction effects of the six factors on the bearing pressure.

First, according to the data presented in Table 3, the goodness of fit for the bearing pressure model is calculated to be 185.40, and the significance level of the model is less than 0.001. The modified model exhibits excellent fit and high reliability. Then based on the data in Table 3, the quadratic polynomial regression equation representing the variation in the bearing pressure with the six characteristic parameters is subsequently fitted as R_1_. Similarly, the prediction models for the coefficient of friction R_2_, for temperature R_3_, and for leakage volume R_4_. Finally, the interaction effects of the six characteristic parameters of the microstructure on the oil film bearing pressure are shown in Fig. 9.

14 $$\begin{gathered} {R_1}=1.665 \times {10^6} - 16338.194A+4572.690B - 67572.346C+58837.281D+ \hfill \\26348.191E+1433.305F+9.12706AB+3.55753AC+4.49960AD - \hfill \\ 13.57677AE+1.34846AF+3.94386BC+26.32513BD - 251.308BE \hfill \\ +1.16113BF - 30.64938CD+1185.399CE+1.51998CF - 310.73113 \hfill \\ DE - 0.769182DF+88.79268EF+24.0112{A^2} - 15.848{B^2}+786.788 \hfill \\ {C^2} - 532.783{D^2} - 15585.185{E^2} - 1.35672{F^2} \hfill \\ \end{gathered}$$

15 $$\begin{gathered} {R_2}= - 0.00643+4.972 \times {10^{ - 6}}A - 2.7 \times {10^{ - 5}}B+1.40 \times {10^{ - 4}}C - 1.6 \times {10^{ - 5}}D - \hfill \\ {\text{ }}4.55 \times {10^{ - 4}}E+2.1 \times {10^{ - 5}}F - 4.578 \times {10^{ - 9}}AB - 4.274 \times {10^{ - 9}}AC - 1.534 \times \hfill \\ {\text{ }}{10^{ - 9}}AD+1.196 \times {10^{ - 7}}AE - 1.405 \times {10^{ - 10}}AF - 7.108 \times {10^{ - 9}}BC - 6.731 \hfill \\ {\text{ }} \times {10^{ - 9}}BD+1.575 \times {10^{ - 7}}BE - 2.355 \times {10^{ - 10}}BF+1.725 \times {10^{ - 8}}CD+1.098 \hfill \\ {\text{ }} \times {10^{ - 6}}CE+1.156 \times {10^{ - 10}}CF - 5.4326 \times {10^{ - 7}}DE - 8.752 \times {10^{ - 9}}DF - 2.91 \hfill \\ {\text{ }}5 \times {10^{ - 10}}EF - 8.006 \times {10^{ - 9}}{A^2}+8.887 \times {10^{ - 8}}{B^2} - 1.8006 \times {10^{ - 6}}{C^2}+2.498 \hfill \\ {\text{ }} \times {10^{ - 7}}{D^2}+4.0 \times {10^{ - 5}}{E^2} - 1.00061 \times {10^{ - 8}}{F^2} \hfill \\ \end{gathered}$$

16 $$\begin{gathered} {R_3}=158.793+0.0032A - 0.1408B+0.2557C+0.4098D+{\text{5}}{\text{0.2997}}E \hfill \\ - 0.1675F - 1.30 \times {10^{ - 4}}AB - 3.9 \times {10^{ - 5}}AC - 9.6 \times {10^{ - 5}}AD - 5.1 \hfill \\ \times {10^{ - 5}}AE - 5.036 \times {10^{ - 6}}AF+2.08 \times {10^{ - 4}}BC - 1.52 \times {10^{ - 4}}BD+2 \hfill \\ 0.395 \times {10^{ - 3}}BE - 3.1 \times {10^{ - 5}}BF+1.3 \times {10^{ - 5}}CD+0.031CE+5.0 \times \hfill \\ {10^{ - 5}}CF - 2.778 \times {10^{ - 3}}DE - 1.01 \times {10^{ - 5}}DF - 1.733 \times {10^{ - 3}}EF - 1.4 \hfill \\ \times {10^{ - 5}}{A^2}+5.83 \times {10^{ - 4}}{B^2} - 5.789 \times {10^{ - 3}}{C^2} - 2.442 \times {10^{ - 3}}{D^2} - 0.4 \hfill \\ 728{E^2}+9.2 \times {10^{ - 5}}{F^2} \hfill \\ \end{gathered}$$

17 $$\begin{gathered} {R_4}=0.03625+7.095 \times {10^{ - 6}}A - 7.323 \times {10^{ - 6}}B+2.850 \times {10^{ - 6}}C+2.4 \times {10^{ - 5}}D+ \hfill \\ {\text{ }}2.049 \times {10^{ - 3}}E - 5.4 \times {10^{ - 5}}F - 1.163 \times {10^{ - 8}}AB+1.088 \times {10^{ - 8}}AC - 6.694 \times \hfill \\ {\text{ }}{10^{ - 9}}AD - 6.757 \times {10^{ - 8}}AE - 1.009 \times {10^{ - 9}}AF+1.955 \times {10^{ - 8}}BC - 5.610 \times \hfill \\ {\text{ }}{10^{ - 9}}BD - 2.100 \times {10^{ - 7}}BE - 3.059 \times {10^{ - 9}}BF+4.915 \times {10^{ - 8}}CD+2.396 \times \hfill \\ {\text{ }}{10^{ - 6}}CE+1.071 \times {10^{ - 8}}CF - 7.221 \times {10^{ - 8}}DE - 1.159 \times {10^{ - 8}}DF - 7.322 \times \hfill \\ {\text{ }}{10^{ - 8}}EF - 8.151 \times {10^{ - 9}}{A^2}+3.847 \times {10^{ - 8}}{B^2} - 4.151 \times {10^{ - 7}}{C^2} - 1.038 \times {10^{ - 7}} \hfill \\ {\text{ }}{D^2} - 2.02 \times {10^{ - 4}}{E^2}+2.785 \times {10^{ - 8}}{F^2} \hfill \\ \end{gathered}$$

The response surface analysis of the interaction effects of six factors two by two on the bearing pressure performance is shown in Table 4. The order of influence of the six factors on the bearing pressure can be determined as: B > A > E > D > C > F. Similarly, the order of influence on the friction coefficient is *B* > *A* > *D* > *E* > *F* > *C*, and the order of influence on the temperature is *B* > *A* > *D* > *E* > *C* > *F*. The order of influence on the leakage is *B* > *A* > *C* > *D* > *F* > *E*.

**Table 4:** Regression equation analysis table of bearing pressure response surface test.

Sources of variance	Oil film bearing pressure R1				
	Sum of squares	Freedom degrees	Mean Squared Value	F	*P*
Model	2.044E + 12	27	7.572E + 10	185.40	< 0.0001
A	1.733E + 11	1	1.733E + 11	424.45	< 0.0001
B	8.407E + 11	1	8.407E + 11	2058.53	< 0.0001
C	6.676E + 09	1	6.676E + 09	16.35	0.0016
D	1.557E + 10	1	1.557E + 10	38.12	< 0.0001
E	2.697E + 10	1	2.697E + 10	66.05	< 0.0001
F	5.663E + 09	1	5.663E + 09	13.87	0.0029
AB	1.382E + 10	1	1.382E + 10	33.85	< 0.0001
AC	1.454E + 08	1	1.454E + 08	0.3560	0.5618
AD	1.261E + 09	1	1.261E + 09	3.09	0.1044
AE	2.870E + 07	1	2.870E + 07	0.0703	0.7954
AF	2.831E + 09	1	2.831E + 09	6.93	0.0219
BC	6.432E + 07	1	6.432E + 07	0.1575	0.6984
BD	1.554E + 10	1	1.554E + 10	38.04	< 0.0001
BE	3.540E + 09	1	3.540E + 09	8.67	0.0123
BF	7.556E + 08	1	7.556E + 08	1.85	0.1988
CD	3.598E + 08	1	3.598E + 08	0.8810	0.3664
CE	1.345E + 09	1	1.345E + 09	3.29	0.0946
CF	2.212E + 07	1	2.212E + 07	0.0542	0.8199
DE	4.418E + 08	1	4.418E + 08	1.08	0.3188
DF	2.707E + 07	1	2.707E + 07	0.0663	0.8012
EF	9.018E + 08	1	9.018E + 08	2.21	0.1631
A^2^	8.643E + 09	1	8.643E + 09	21.16	0.0006
B^2^	4.880E + 08	1	4.880E + 08	1.19	0.2958
C^2^	9.280E + 08	1	9.280E + 08	2.27	0.1576
D^2^	6.809E + 09	1	6.809E + 09	16.67	0.0015
E^2^	3.641E + 07	1	3.641E + 07	0.0892	0.7703
F^2^	2.759E + 07	1	2.759E + 07	0.0676	0.7993
Residual	4.901E + 09	12	4.084E + 08		
Lack of fit	4.901E + 09	7	7.001E + 08		
Pure error	0.0000	5	0.0000		
Sum	2.049E + 12	39			

The micro-texture parameters *A*, *B*, *C*, *D*, *E*, and *F* are selected as the optimization parameters, with the maximum oil film bearing pressure, minimum friction coefficient, minimum temperature, and minimum leakage as the objectives. Constraints are set such that the bearing pressure exceeds that of the untextured surface, whereas the friction coefficient, leakage, and temperature rise are less than those of the untextured surface. These conditions are used to establish a multi-objective optimization mathematical model, represented by Eq. (18).

18 $$\begin{gathered} \hbox{min} F(P)={[ - {R_1}, {R_2}, {R_3}, {R_4}]^T} \hfill \\ S.T\left\{ {\begin{array}{*{20}{c}} {{f_1}(P) \geq 13710337} \\ {{f_2}(P) \leq 108.95} \\ {{f_3}(P) \leq 0.00446} \\ {{f_4}(P) \leq 0.0173} \\ {P=\left[ {A,B,C,D,E,F} \right]} \\ {200 \leq A \leq 400} \\ {90 \leq B \leq 210} \\ {30 \leq C \leq 50} \\ {35 \leq D \leq 75} \\ {4^\circ \leq E \leq 6^\circ } \\ {900 \leq F \leq 1100} \end{array}} \right. \hfill \\ \end{gathered}$$

**Fig. 10:**
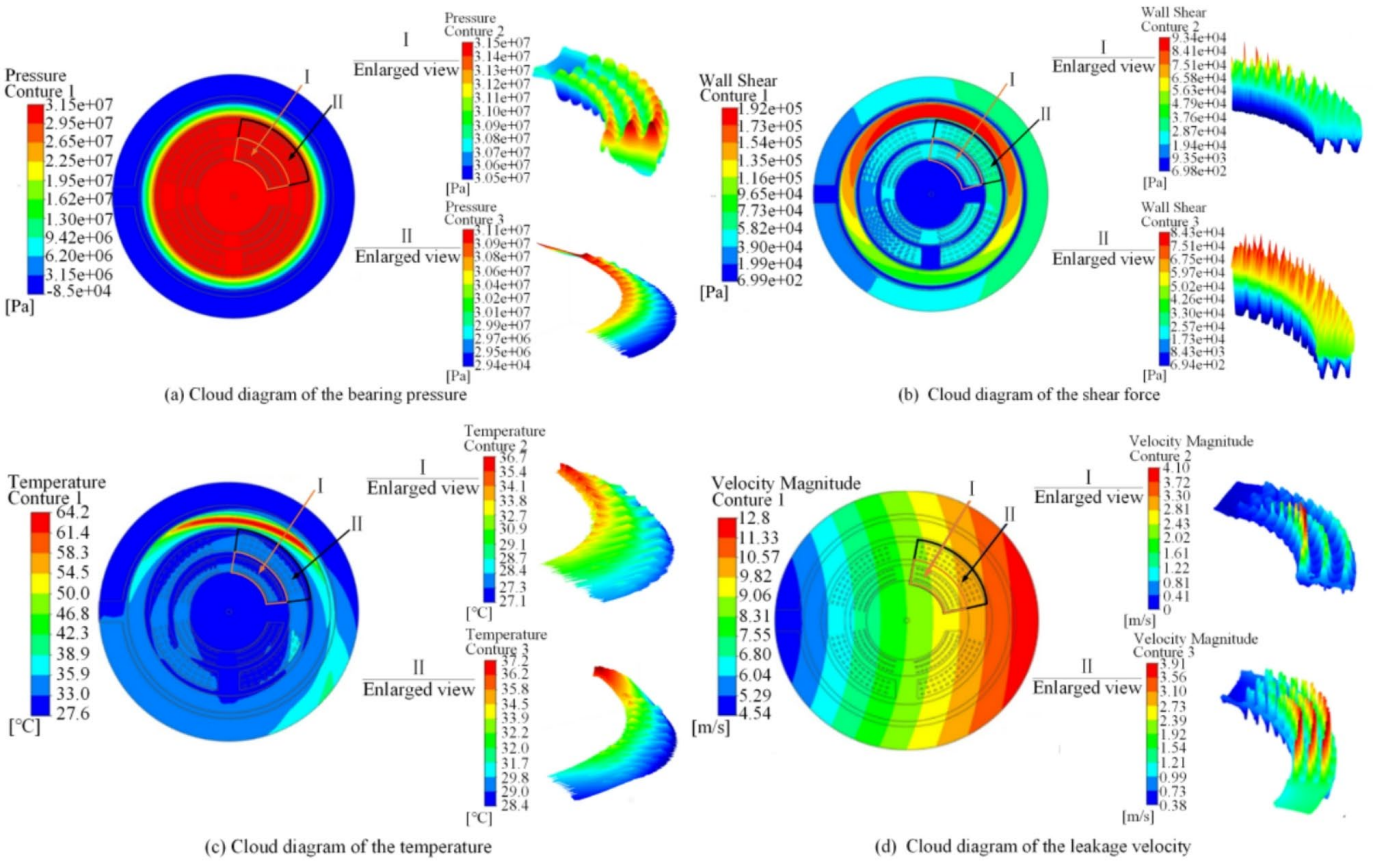
Cloud diagram of the optimal texture scheme.

The optimal parameter solutions are as follows: A = 372.251 μm, B = 201.836 μm, C = 48.148 μm, D = 74.247 μm, E = 5.846°, and F = 1089.759 μm. After rounding, the values are A = 372 μm, B = 202 μm, C = 48 μm, D = 74 μm, E = 5.9°, and F = 1090 μm. By utilizing these optimal micro-texture parameters, the oil film model of the textured axial piston pump slipper pair was reconstructed and simulated, with the corresponding performance cloud diagram depicted in Fig. 10. The bearing pressure of the oil film for the EOOPT slipper pair, calculated using Eqs. (9) to (13), is 16410637.32 Pa, which represents a 19.69% increase compared with that of the prototype. The friction coefficient is 0.00352, which is 21.08% reduction from the prototype. The average temperature is 45.3 °C, which is14.20% lower than that of the prototype, and the leakage rate is 0.01483 L/s, which is 14.03% decrease from the prototype.

## Experimental research

**Fig. 11:**
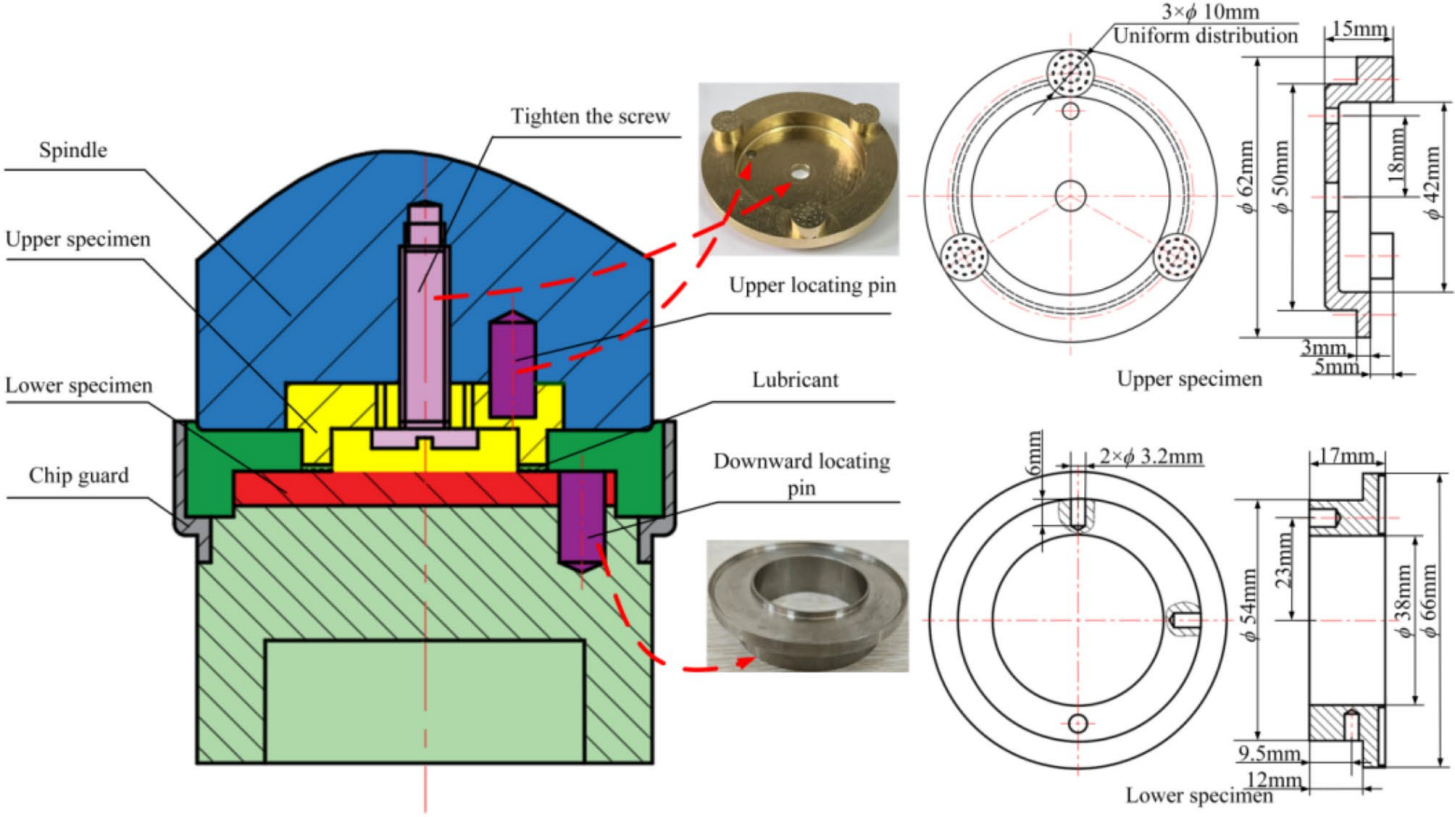
The upper and lower sample diagrams of the simulated slipper pair.

The upper and lower samples of the simulated slipper friction pair are designed as depicted in Fig. 11. The upper specimen features three platforms with a diameter of Φ10 mm, which serve to mimic the slipper, whereas the annular groove between the lower specimen’s Φ66 mm and Φ33 mm diameters is designed to represent the upper surface of the swash plate. The upper specimen material is H62 brass, and the lower specimen material is 316 L stainless steel. Prior to micro-texturing processing, the material surfaces were ground and polished, followed by ultrasonic cleaning and drying to remove surface contaminants. The surface hardness of the treated upper and lower specimens is 79HV and 187HV, respectively, and the surface roughness is 4.42 ± 1.34 μm and 4.89 ± 1.25 μm, respectively. During the friction wear experiment, the annular groove can be filled with hydraulic oil to simulate the operational conditions of the slipper pair in an oil-rich environment.

Micro-texturing is prepared using the SmartCNC500E_DRTD five-axis machining center as shown in Fig. 12①. First, the specimen is fixed on the worktable using a custom-designed fixture, and the micro-texture unit cell model file created with three-dimensional modeling software is imported. The model file is then programmed and the machining path is controlled using the machining center’s built-in program system. The tool entry method is set to a closed-path spiral toolpath, and the feed rate is set to 300 mm/min. The machining center’s X, Y, and Z-axis motion positioning accuracies are 0.008 mm, 0.006 mm, and 0.006 mm, respectively, and the X, Y, and Z-axis repeat positioning accuracies are 0.005 mm, 0.005 mm, and 0.005 mm, respectively. Finally, a tungsten carbide ball-end micro-mill with a diameter of 0.1 mm is used to fine-mill the specimen surface along the pre-set machining path, resulting in the micro-texture profile as shown in Fig. 13.

The grinding experiments on both the upper and lower samples were conducted using the MMW-1 A vertical friction wear testing machine, as depicted in Fig. 12②. To guarantee that the oil film pressure during the friction and wear tests matches the initial pressure conditions established by the simulation, the spindle speed is adjusted to 600 r/min, and the downward pressure exerted by the spindle is set to 150 N. Prior to the test, the upper and lower samples were subjected to ultrasonic cleaning and then dried with compressed air before being installed in the designated fixtures. The spindle speed is incrementally increased from 0 r/min to 600 r/min and allowed to idle for 10 min to ensure operational stability. Throughout the test, the upper sample remains in a rotating state (simulating the operational condition of the axial plunger pump slipper), whereas the lower sample is held stationary (mimicking the operational condition of the axial plunger pump swash plate). The lubricating oil used in the experiment has a density *ρ* of 872.5 kg/m³ and a dynamic viscosity *η* of 1.553 Pa·s. Each set of experiments lasts for 900 s, with each set repeated 5 times to obtain friction coefficient and oil film temperature data for subsequent experimental verification and analysis.

**Fig. 12:**
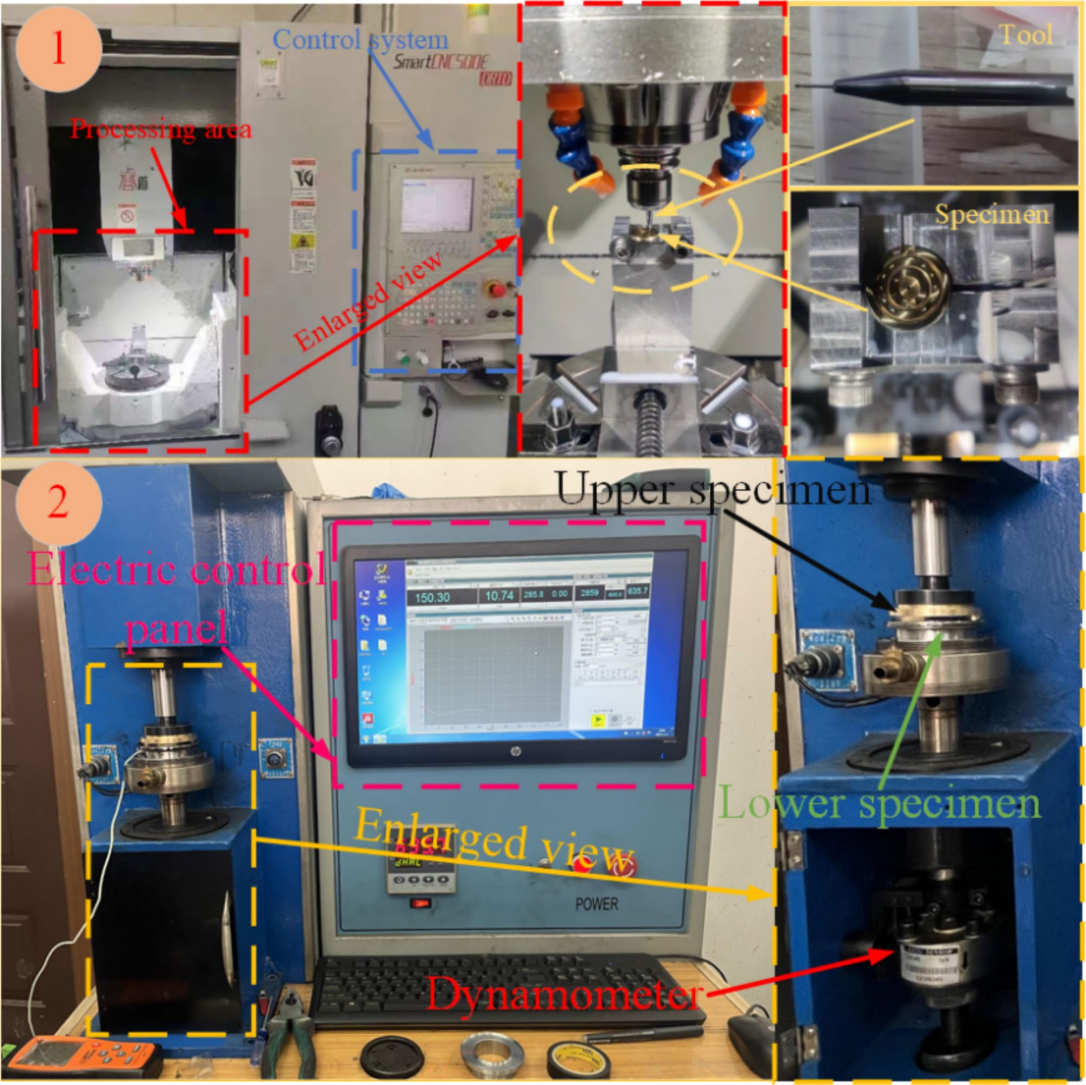
Machining center and friction and wear testing machine.

**Fig. 13:**
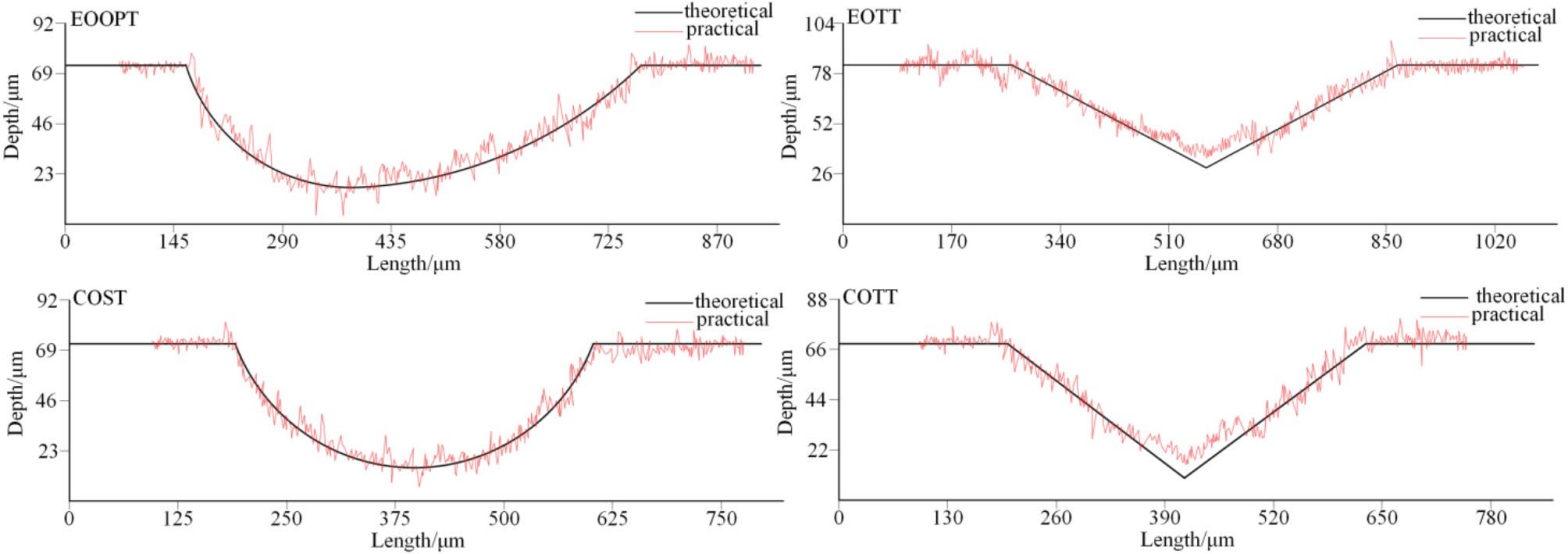
Comparison of practical micro texture profile with the theoretical profile.

**Fig. 14:**
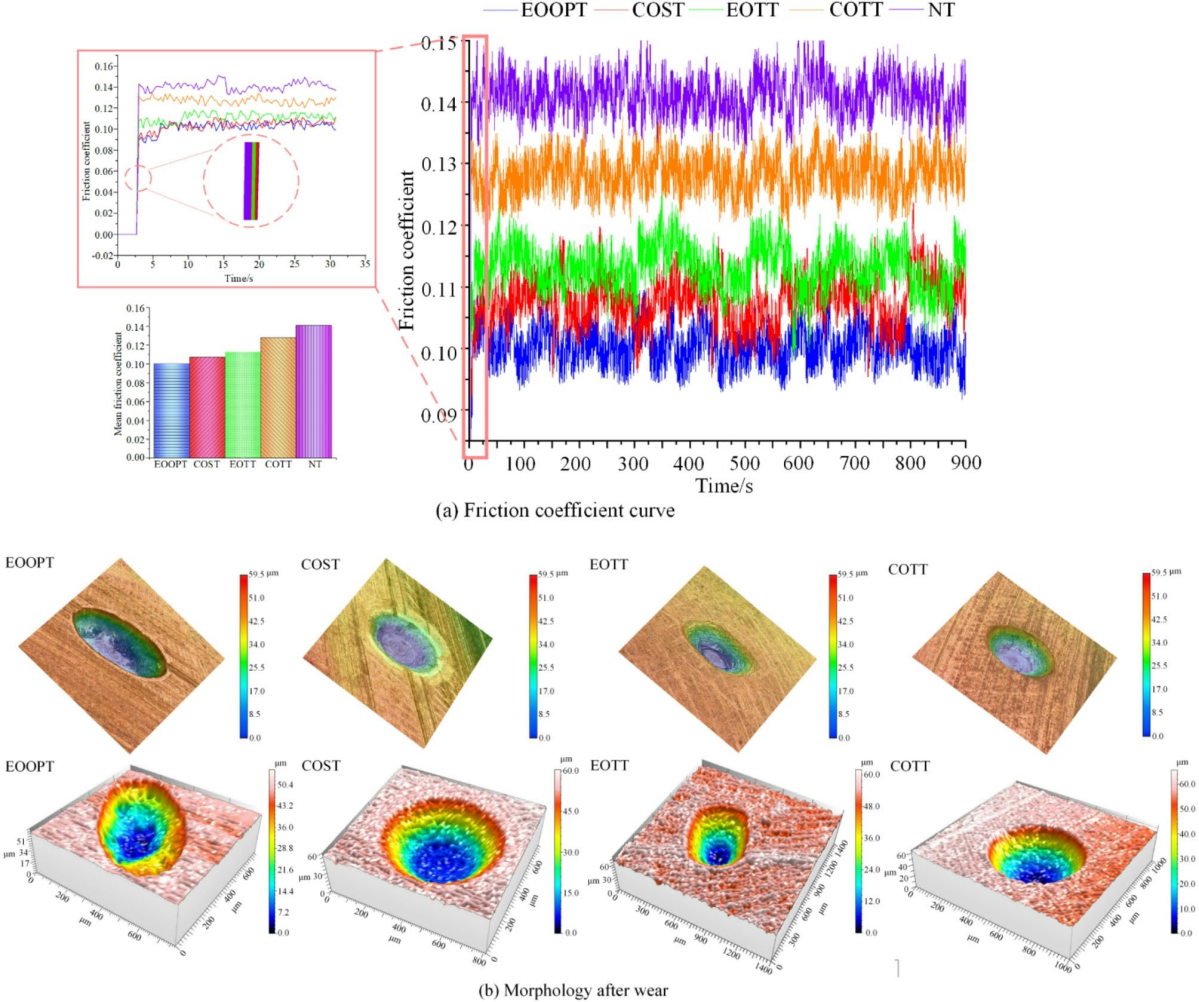
Wear test results.

The upper sample of the simulated slipper and the lower sample of the simulated swash plate were meticulously designed and manufactured. The upper sample has four distinct textures, as outlined in Table 1. The data and laws derived from the friction experiment are compared with previous simulation analysis results to validate the accuracy of the simulation method and its conclusions. Upon completion of the friction wear experiment, the friction coefficient curve is depicted in Fig. 14a. The surface morphology of the sample was evaluated using the VHX5000 ultra-depth-of-field microscopy and the NANOVEA PS50 non-contact surface profilometer, with the results presented in Fig. 14b.

As depicted in Fig. 14a, the friction coefficient of the oil film for the slipper pair is consistent with the simulation analysis presented in “Experimental research”, specifically: EOOPT > COST > EOTT > COTT > NT. When the wear morphology in Fig. 14b is examined, it becomes apparent that the NT sample surface exhibits distinct scratches, whereas the COTT sample surface shows noticeable changes in texture edge morphology. The EOTT sample displays more secondary wear on its surface, leading to granular damage, and the overall surface morphology of the COST sample is slightly compromised. In contrast, the EOOPT sample features shallow wear scratches and maintains a complete micro-texture morphology. These findings further substantiate that micro-texturing is advantageous for reducing the friction coefficient of the slipper pair and that, compared with other textures, EOOPT has superior anti-friction properties.

**Fig. 15:**
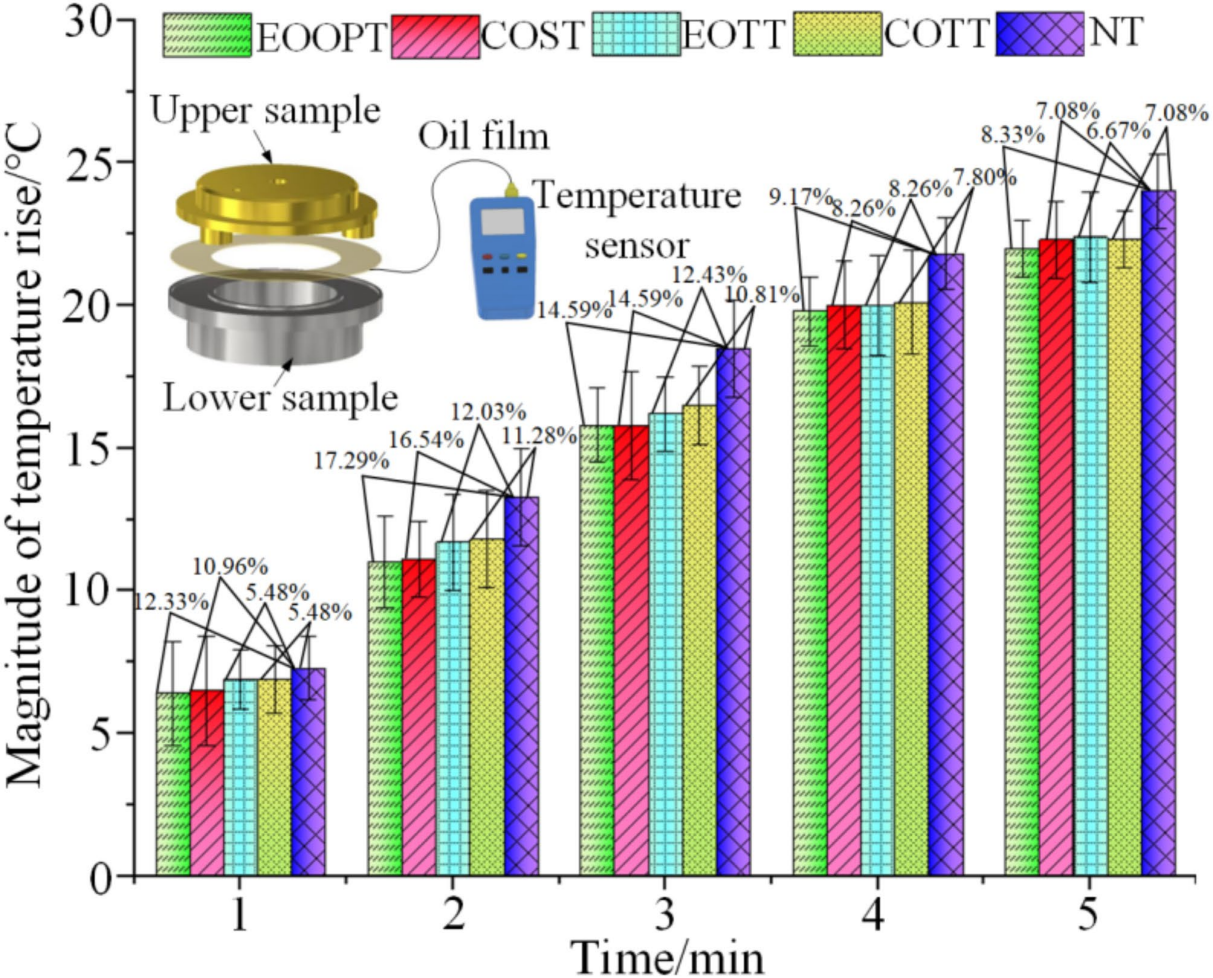
Temperature rise curve with time.

In the course of the friction wear experiments conducted on each friction pair, temperature measurements were taken at 60-second intervals, with each set of trials being replicated five times. The temperature fluctuations of the various friction pairs are depicted in Fig. 15. Throughout the experimental process, the temperature of the friction subsurface gradually increased, with the amplitude of this increase becoming progressively more pronounced. Regardless of whether the average temperature or the temperature at any specific moment is considered, the textured sample demonstrated a lower temperature increase than did the non-textured sample. Notably, EOOPT exhibited the least temperature increase, followed by COST, EOTT, and COTT. The primary reason for this phenomenon is that the micro-texture enhances the contact area between the sample and the lubricating oil, thereby facilitating the formation of a more stable lubricating oil film. The lubricating oil stored within the texture provides an additional lubrication layer, promoting more effective thermal dissipation and reducing the heat generated by surface friction. In scenarios with identical depths and opening areas, the EOOPT texture is more effective at accommodating lubricating oil than other texture shapes are, ensuring a smoother flow of lubricating oil and thereby reducing the rate of temperature increase. The relationship between the friction temperature and wear resistance of different micro-texture pairs corroborates the accuracy of the simulation analysis of the friction performance of the micro-texture pairs. Furthermore, micro-texturing is advantageous for reducing the friction coefficient of the slipper pair, with EOOPT demonstrating superior anti-friction properties compared with those of other textures. The experimental results are consistent with the results obtained from simulation analysis, and they verify each other, proving the correctness of the research method.

## Conclusion

To increase the wear resistance of the slipper pair in the axial piston pump, reduce heat generation and leakage during operation, and meet the demands of precise working conditions, the impact of micro-texturing on the piston pump was investigated using computational fluid dynamics and response surface methodology. The findings were subsequently validated through experimentation. The conclusions are as follows:

1. On the basis of the structural characteristics of the slipper friction pair in the swash plate axial piston pump, a dynamic and hydrodynamic performance analysis model of the oil film on the slipper surface was established. This model provides a theoretical foundation for subsequent analyses of bearing performance, friction and wear resistance, temperature rise characteristics, and sealing performance. By comparing the effects of four types of micro-textures and fifteen circular ring surface combination distribution schemes on surface oil film performance, it was determined that the Elliptic Opening Offset Parabola Micro Texture (EOOPT) is the optimal texture type, and auxiliary support belts 1 and 2 are the most effective texture positions. By comparing the effects of four types of micro-textures and fifteen kinds of texture ring shore combination distribution schemes on the oil film performance on the slipper surface, the EOOPT is determined to be the best texture type, and the inner auxiliary support zones 1 and 2 are the best texture positions.
2. Upon optimizing the position distribution and structural size parameters of EOOPT, the optimal texture parameters for slippers are identified as follows: A = 400 μm, B = 211 μm, C = 50 μm, D = 75 μm, E = 5.9°, and F = 1090 μm. For these parameters, the bearing capacity of the slipper pair is increased by 19.69% in comparison with the optimal parameters of a non-woven slipper pair. Furthermore, the friction coefficient, average temperature increase, and leakage are reduced by 21.08%, 14.20%, and 14.03%, respectively. This optimization maximizes the potential of EOOPT to improve the surface working performance of slippers.
3. The results of the friction and wear experiments substantiate the validity of the theoretical and simulation analyses. The examination more accurately depicts the effect of micro-textures on the functioning of the friction pair and delineates the mechanism through which micro-textures augment the performance of the slipper.

## Data Availability

The authors declare that the data supporting the findings of this study are available within the paper. More data that needs to be obtained will be available upon reasonable request by contacting the corresponding author Jing Luo at ling19991202@163.com.
